# Data from Emergency Medical Service Activities: A Novel Approach to Monitoring COVID-19 and Other Infectious Diseases

**DOI:** 10.3390/diagnostics15020181

**Published:** 2025-01-14

**Authors:** Daniele del Re, Luigi Palla, Paolo Meridiani, Livia Soffi, Michele Tancredi Loiudice, Martina Antinozzi, Maria Sofia Cattaruzza

**Affiliations:** 1 Department of Physics, Sapienza University of Rome, 00185 Rome, Italy; daniele.delre@uniroma1.it; 2 Department of Public Health and Infectious Diseases, Sapienza University of Rome, 00185 Rome, Italy; mariasofia.cattaruzza@uniroma1.it; 3 INFN Istituto Nazionale Fisica Nucleare, Sezione di Roma, 00146 Rome, Italy; paolo.meridiani@roma1.infn.it (P.M.); livia.soffi@roma1.infn.it (L.S.); 4 Department of Developmental and Social Psychology, Faculty of Medicine and Psychology, Sapienza University of Rome, 00185 Rome, Italy; michele.loiudice@uniroma1.it

**Keywords:** COVID-19, emergency medical services, pandemic, excess mortality estimation, infectious diseases

## Abstract

**Background**: Italy, particularly the northern region of Lombardy, has experienced very high rates of COVID-19 cases and deaths. Several indicators, i.e., the number of new positive cases, deaths and hospitalizations, have been used to monitor virus spread, but all suffer from biases. The aim of this study was to evaluate an alternative data source from Emergency Medical Service (EMS) activities for COVID-19 monitoring. **Methods**: Calls to the emergency number (112) in Lombardy (years 2015–2022) were studied and their overlap with the COVID-19 pandemic, influenza and official mortality peaks were evaluated. Modeling it as a counting process, a specific cause contribution (i.e., COVID-19 symptoms, the “signal”) was identified and enucleated from all other contributions (the “background”), and the latter was subtracted from the total observed number of calls using statistical methods for excess event estimation. **Results**: A total of 6,094,502 records were analyzed and filtered for respiratory and cardiological symptoms to identify potential COVID-19 patients, yielding 742,852 relevant records. Results show that EMS data mirrored the time series of cases or deaths in Lombardy, with good agreement also being found with seasonal flu outbreaks. **Conclusions**: This novel approach, combined with a machine learning predictive approach, could be a powerful public health tool to signal the start of disease outbreaks and monitor the spread of infectious diseases.

## 1. Introduction

The health emergency caused by the spread of coronavirus disease 2019 (COVID-19), firstly in China and then worldwide, suddenly became a global emergency and was then declared a pandemic by the World Health Organization (WHO) on the 11th of March 2020 [1]. After three years of emergency and major health and economic burdens, the pandemic was then declared to be ceased on the 5th of May 2023 [2]. However, despite over 775 million confirmed cases, and more than 7 million deaths officially recorded in the world, COVID-19 did not result in the worst recorded pandemic, partly thanks to the success of public health efforts in promptly responding to the virus outbreak globally [3].

The North of Italy, the first country to be hit in Europe, was the first affected area in the country, with very high rates of cases and deaths. Across Europe, Italy is the third country in terms of a cumulative number of cases with more than 26 million infected people after France and Germany, with 39 and around 38 million, respectively. According to WHO’s Coronavirus Dashboard, as of 20th June 2023, after the pandemic was officially declared to be over, Italy ranked first for mortality in the European Region, with more than 195,000 cumulative deaths, followed by Germany, with more than 175,000, and France, with approximately 168,000 [4].

Soon after the COVID-19 pandemic became apparent in Italy (February 2020), the Italian government declared a nationwide lockdown on the 9th of March 2020 (which ended on the 3rd of May 2020) in order to prevent the healthcare system from collapsing under the weight of the rapid spread of the disease, which was causing thousands of contagions and deaths in a very limited period of time [5]. Thanks to this public health policy, Italy was then able to limit the COVID-19 spread, and the healthcare system could at least try to cope with the emergency; nonetheless, health professionals had to face hard therapeutic choices due to the lack of medical devices and, despite the measures taken, they had to carry a large burden, with 250,000 confirmed cases and 35,000 deaths in that first 2020 wave [6].

During the pandemic, there was a big concern about the best choice of surveillance system to monitor COVID-19, as the impact of accurate assessment of data was recognized as being crucial to controlling the virus spread. Moreover, the reliability of the data source was also considered as an essential aspect in regard to informing both the public and the policymakers, and it particularly helped to develop adequate public health policies to control the pandemic both at the individual and population levels [7].

In this context, the data used to control the spread of the pandemic came from a variety of sources, such as hospital or local healthcare authority datasets that reported the count of COVID-19-positive cases, the number of infected and hospitalized patients, patients admitted to intensive care units (ICUs), as well as death counts. It is important to bear in mind that any surveillance system used has pros and cons that public health professionals need to consider while interpreting data. For example, daily collected COVID-19 new positive cases depend on the swab tests administered to the population, which is linked at the same time to the availability of those tests as well as to the prevalence of the disease in that population [8]. In addition, unreported cases (e.g., paucisymptomatic patients not reaching the healthcare authorities) must be taken into account, and their number usually increases as the contagiousness of the disease increases in parallel with the reduction in symptoms, which can be caused by changes in the virulence due to the natural development of the virus as well as by the introduction of new drugs or vaccines to tackle the disease, making the data less reliable. Another source of data for surveillance systems can be data coming from mortality analyses, as with seasonal influenza surveillance [9,10,11]. These data may be considered as an alternative and reliable source also for viruses such as COVID-19 when the identification of the specific COVID-19 mortality contribution is reliable. Indeed, excess mortality has been used in Italy [12,13,14,15,16] and other countries [17] as a marker of virus diffusion among the population, and it has also been used to compare mortality across countries [18,19], even if most studies focused just on the first COVID-19 wave throughout 2020 [13,14,15,16,19,20]. Despite their wide use, mortality data have the disadvantage of death occurring several days after the day of diagnosis, so they lag behind the actual spread of the disease and are therefore an untimely detection tool.

Nowadays, even after the end of the COVID-19 pandemic, we still need to monitor COVID-19 diffusion in the population, as we do for seasonal influenza, since it is essential to promptly track the possible onset of new virus variants and to alert public health policymakers.

In light of all the above considerations, the aim of this study was to evaluate an alternative source of data coming from the Emergency Medical Service Activities to monitor the prevalence of COVID-19. In fact, we used the calls to the 112 emergency number in the Lombardy region for the years 2015–2022 and studied their overlap with COVID-19 pandemic and influenza peaks. Emergency call data have been investigated before, mostly but not exclusively in relation to COVID-19, both in Italian regions and in other countries or regions, albeit for different purposes, i.e., to determine the impact of COVID-19 on the propensity to use and the reason for use of emergency services [21,22,23] or to predict the number of intensive care admissions [24,25], especially in the first period (2019–2020) of the pandemic. Here, we propose using emergency call data as a signal for early detection of future pandemic waves or of other extraordinary health events, with a novel application to the Lombardy emergency call time-series (2015–2022) of excess estimation methodology [26].

## 2. Materials and Methods

Data used in this paper come from the calls to the number 112 in the Lombardy region in Italy from 1st January 2015 to 30th April 2022. These data were provided by the EMS AREU (Azienda Regionale Emergenza/Urgenza) mission registry. The organization structure of AREU is composed of four Regional Emergency Health Operations Rooms of inter-provincial significance (Italian acronym is SOREU, which stands for “Sale Operative Regionali dell’Emergenza Urgenza sanitaria a valenza interprovinciale”); they manage medical emergency calls by sending the most appropriate means (such as specific healthcare professionals and/or emergency vehicles) until the rescue is completed and/or the patient is eventually entrusted to the most suitable hospital facilities.

In all of the four SOREU, a homogeneous organizational model is adopted, with common procedures being used to create a single regional system capable of achieving real collaboration and operational integration in the border territories [27].

The four SOREU cover all territories of the Lombardy region and are named and based as follows:

(1) SOREU “Metro” (Metropolitan) in Milan;
(2) SOREU “Laghi” (Lakes) in Como;
(3) SOREU “Alpina” (Alpine) in Bergamo;
(4) SOREU “Pianura” (Plain) in Pavia.

All EMS records are registered in the SaS-AREU portal (SAS Institute, Cary, NC, USA). The portal contains all data related to emergency calls, logistical elements and the clinical information of the patient. We use all the information retrieved from the call; in fact, during the medical emergency call, the 112 operator identifies the priority dispatch level by using a decision tree. In this way, all data correlated with the calls are collected in a standardized way. We selected all patients with symptoms falling under the respiratory inflammatory tree and cardiological diseases [28,29].

The database exploited in this study includes several different pieces of information for each call. The most relevant ones used to characterize patients affected by COVID-19 are listed below:

- Issue of the call (e.g., breathing difficulty, chest pain or heart disease, neurological disorder, musculoskeletal disease, intoxication or drug overdose, accidents and other major or minor injuries);
- Age;
- Sex;
- Severity evaluated by the triage classification in loco (ascending scale by gravity: white, green, yellow, red);
- Day of the call;
- Hour of the day of the call;
- Code evaluated by the trauma triage and scoring at the arrival of the patient in the hospital (ascending scale by gravity: white, green, yellow, red, black);
- Time passed between the ambulance’s departure from the hospital and its arrival to the patient location;
- Time passed between the ambulance arrival to the patient location and its return, i.e., its arrival to hospital.

AREU data were compared with the official number of positive COVID-19 cases in Lombardy [2] and with the mortality data provided by the Italian National Statistics Institute (ISTAT Istituto Nazionale di Statistica) publicly available on their site [30], which represents a national unbiased and reliable source of information. These mortality statistics are produced by ISTAT analysis data submitted to the Civil Status Offices of the municipalities by National Health Service (NHS) doctors. The software used in this study to perform the statistical analysis of these data is ROOT version v6.26.06 [31,32].

The contribution related to a specific cause (e.g., to symptoms related to COVID-19) is separated from all other contributions via the discrimination of “signal” and “baseline” components in a counting experiment. The specific cause we are interested in represents the signal, while any other possible cause of symptoms is the baseline.

This method was already used for analyzing COVID-19 data, and details are described in a previous article [26]. Below, we outline the main aspects.

Using statistical methods based on a maximum-likelihood fit, the baseline contribution is subtracted from the total observed number of calls. The baseline is estimated using the following regression model:

(1) $$b\left[i\right]=offset\;*\left(1+slope*i\right)+amplitude*cos\left(\left(i+phase\right)*\;2\pi /365\right)$$

where *b*[*i*] is the estimated number of calls for day *i* from 1 January 2015 to 30 November 2019; the seasonal behavior is parameterized by a cosine curve with a period of 1 year. The parameters “amplitude”, “phase” and “offset” are estimated from the data. The “offset” is multiplied by a linear function to account for possible year-dependent variations in the number of calls due to changes in the age structure of the population. These parameters have been obtained from periods which do not overlap with influenza and heat waves, i.e., April–May and September–November of 2015, 2016, 2017, 2018 and 2019.

This baseline fitted contribution has been then interpolated to the period of interest (e.g., a COVID-19 wave) and subtracted to obtain the excess of calls:

(2) $$excess\left[i\right]=n\left[i\right]-b\left[i\right]$$

where *n*[*i*] is the number of calls for day *i*.

The uncertainties on *n*[*i*] correspond to the Poissonian error (i.e., *sqrt*(*n*[*i*])) in quadrature with the statistical uncertainty of the estimation of *b*[*i*].

To numerically calculate the strength of the association, we computed a Pearson linear correlation coefficient at different lags between the time series of excess calls to emergency services and the one of excess mortality based on national statistics data.

In addition, the same approach for the baseline subtraction has been repeated for each of the different quantities under study, which were previously listed. In this case, the number of calls is not binned as a function of the day (*i* index, as discussed in the above equations) but, instead, as a function of the variable of interest (e.g., age of the patient, time of the call, etc.). The shape of the baseline contribution still comes from the period used to derive the seasonal cosine-like behavior, while its normalization takes into account the integral of *b*[*i*] in the time period under study (for instance, a specific wave of COVID-19).

In particular, in this analysis, three different types of contributions were identified and studied separately, baseline, flu and COVID-19, and three ranges in time were identified:

- Period 1: 1 July 2016–11 October 2016 and 10 February 2017–20 May 2017. During this period, looking at the time series, flu contribution should be negligible. This period was used to model the baseline;
- Period 2: 15 December 2016–14 February 2017. Here, the peak related to the 2017 flu was very evident;
- Period 3: 11 March 2020–31 March 2020. In this period, the first COVID-19 wave was dominant.

## 3. Results

A total of 6,094,502 records have been analyzed. Preliminarily, the dataset has been filtered, retaining only respiratory and cardiologic issues, thus highly enhancing the sample of COVID-19-related patients. After this, a total selection of 742,852 records are left (roughly 12% of the total).

**Figure 1:**
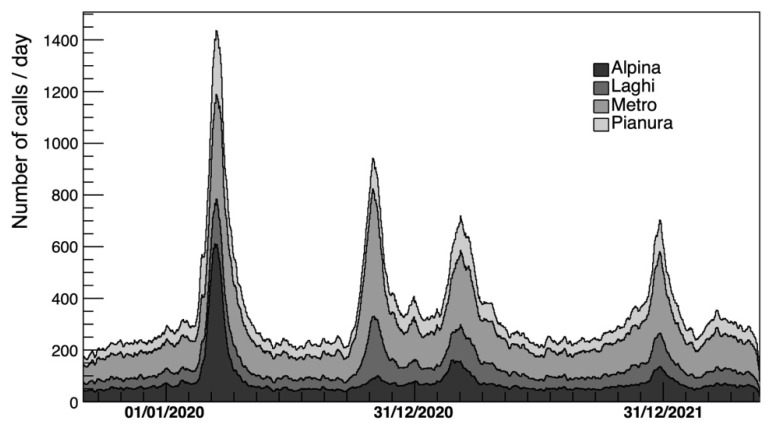
Number of calls per day (weekly averaged) for respiratory and cardiologic issues as a function of time for the different SOREU areas in Lombardy.

Figure 1 shows the time-series of the weekly average number of calls to the Emergency Medical Services as a function of the calendar day in the four different Lombardy SOREU areas (i.e., Alpina, Laghi, Metro and Pianura) before the baseline subtraction.

**Figure 2:**
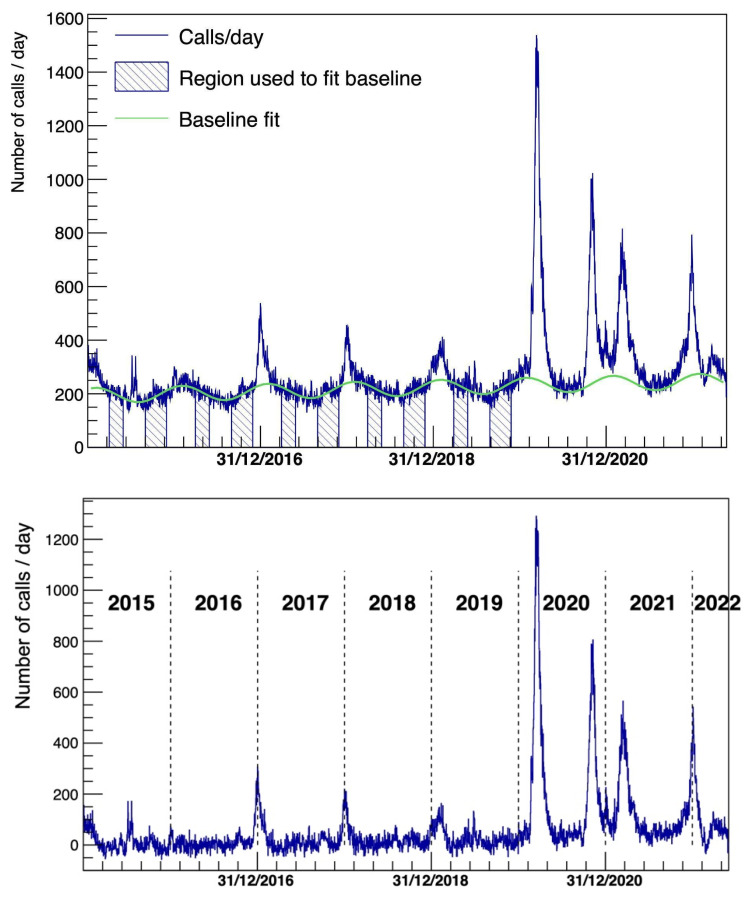
**Top**: Number of calls per day for respiratory and cardiologic issues as a function of time. In green and overlaid, with the baseline contribution being fitted with the parameterization described in the text. **Bottom**: The same as above, but with the subtraction of the fitted baseline contribution.

Figure 2 shows the time series after analyzing respiratory and cardiologic issues from the last 6 years and the baseline-subtracted distribution, following the method discussed in the previous section.

### 3.1. Analysis of the Time Series and Comparison with Official Data and ISTAT Mortality

**Figure 3:**
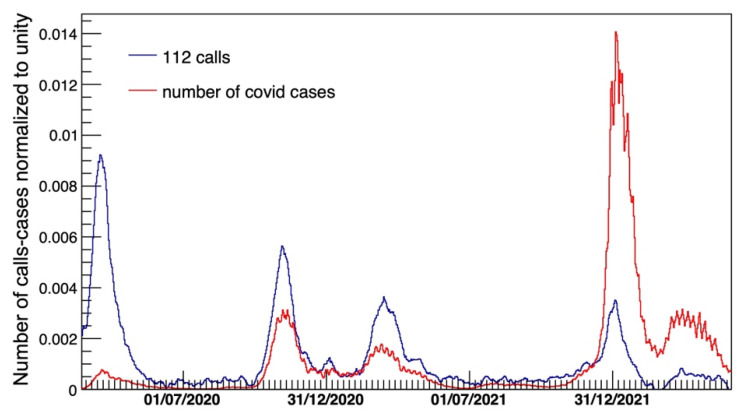
Number of calls per day (weekly averaged) for respiratory and cardiologic issues as a function of time and subtracted from the fitted baseline contribution compared with the official number of positive COVID-19 cases in Lombardy (red line).

Figure 3 shows the time pattern of the COVID-19 contribution outlining the correlation with the official monitoring of the epidemic’s waves [2] based on the daily base counting of COVID-19 positive patients. The distribution of the number of calls to 112 is overlaid with the number of official positive cases.

**Figure 4:**
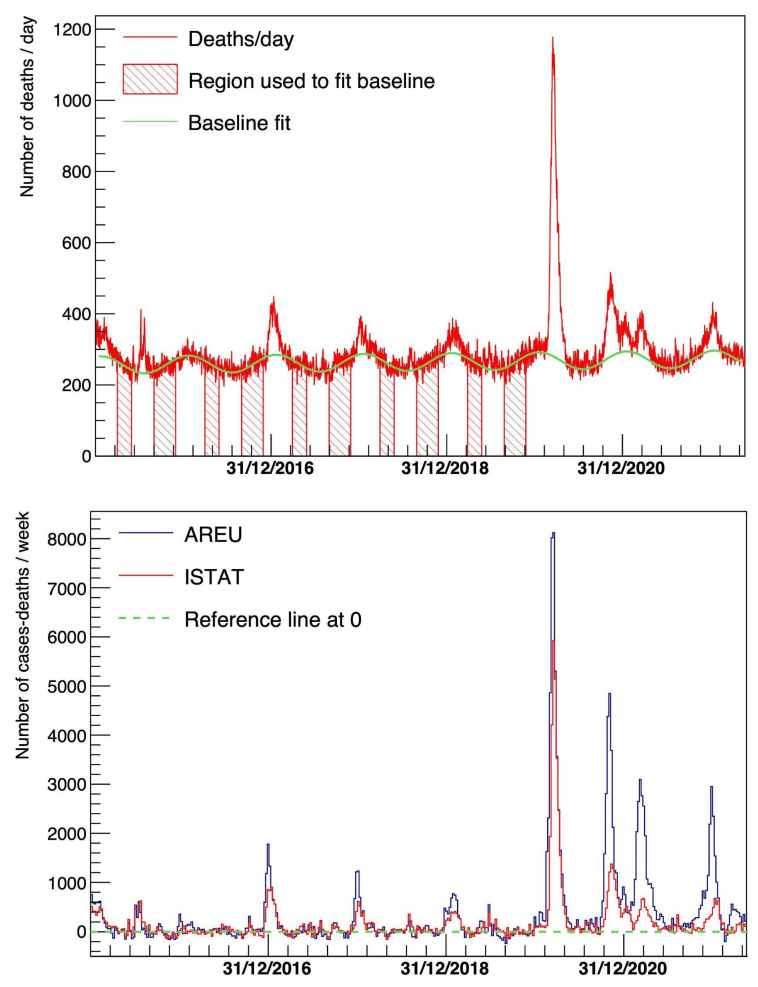
**Top**: Number of deaths per day for Lombardy from ISTAT. Overlaid, baseline contribution fitted with the parameterization described in the text. **Bottom**: Comparison of the baseline-subtracted distributions of the number of calls for respiratory and cardiologic issues per week from AREU and the number of deaths from ISTAT.

Also, the comparison of the AREU data with the COVID-19 mortality excess based on ISTAT mortality data shows great concordance. The fitted distribution for ISTAT mortality data is shown in Figure 4 (top), whereas the two subtracted distributions, from ISTAT and AREU, are compared in Figure 4 (bottom). It can be noted how the peaks relative to the seasonal flu (in years prior to COVID-19 epidemic) and the different COVID-19 waves are visible and match each other for both AREU and ISTAT. We calculated the Pearson linear correlation coefficients between the two time-series displayed in Figure 4 (bottom), separating the COVID-19 period (2020–2022) from the previous period (2015–2019) and found that the contemporary data yielded r = 0.82 and 0.64, respectively, while the maximum of the correlation coefficient was reached for a lag of 6 days for 2020–2022 (r = 0.88) and with a lag at 2 days in the period 2015–2019 (r = 0.67).

### 3.2. Analysis of the Emergency Medical Services Variables

**Figure 5:**
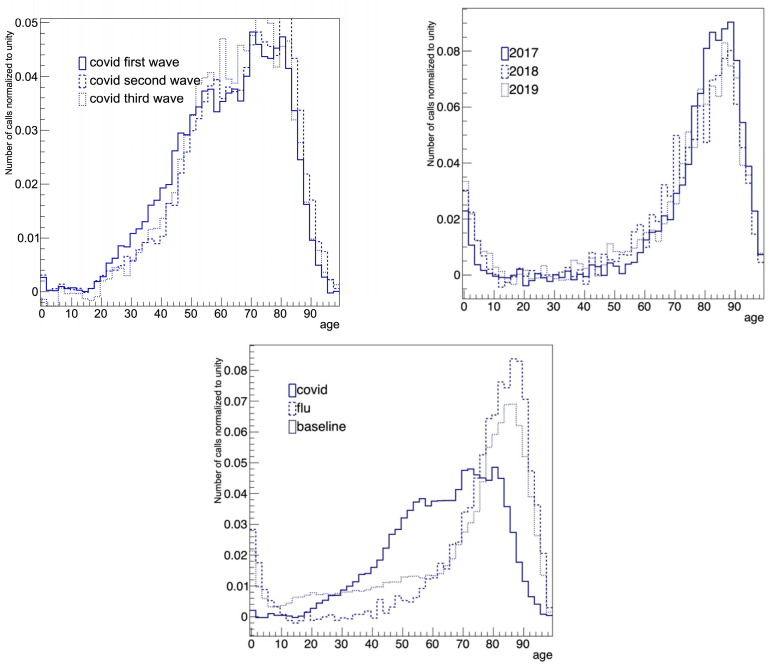
**Top**: Comparison of the baseline-subtracted distributions of the age of the patients for (**left**) the three COVID-19 waves and (**right**) flu for different years. **Bottom**: Comparison of the baseline-subtracted distributions for COVID-19 (period 3), flu (period 2) and baseline (period 1).

Each variable was studied to verify its stability as a function of time and its discriminating power by comparing the baseline, flu and COVID-19 levels. The top of Figure 5 shows the comparison of the baseline-subtracted distributions of patients’ ages for different COVID-19 waves (top left) and for flu in different years (top right). The bottom of Figure 5 compares the baseline (period 1), flu (period 2) and COVID-19 (period 3) levels. The COVID-19 age distribution looks very different from the other two distributions.

**Figure 6:**
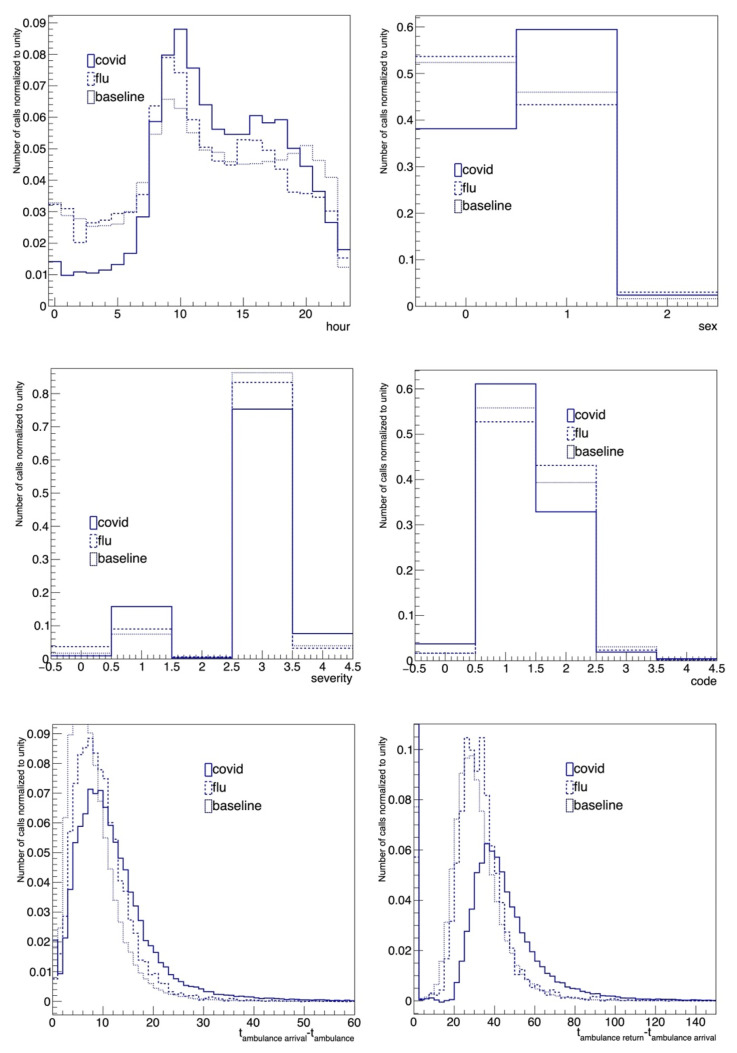
Comparison of the baseline-subtracted distributions of the patients for COVID-19 (period 3), flu (period 2) and baseline (period 1). The top right shows sex (0 = female, 1 = male, 2 = undefined), while the top left shows the hour of the day of the call. The middle left shows the severity of the call (triage coding at the site of rescue: 0 = white, 1 = green, 2 = green/yellow, 3 = yellow, 4 = red), the middle right shows the code of the call (triage coding in hospital: 0 = white, 1 = green, 2 = yellow, 3 = red, 4 = black). The bottom left shows the time passed between the ambulance leaving the hospital and its arrival to the patient location, and the bottom right shows the time passed between the ambulance arrival to the patient location and its arrival to hospital.

The additional variables: disease severity, hour of the day, code of the call, ambulance arrival and return timing have also been analyzed with the same approach. Some examples are shown in Figure 6.

## 4. Discussion

Infectious disease control is one of the public health fields in which correct estimates, in terms of deaths or cases, are paramount in order to contain the dissemination of the disease. However, especially during pandemics, when the situation changes rapidly and there is a large flow of scientific data being reported and publicized, it is not always possible to collect accurate, precise and reliable disease estimates [33]. In particular, during the recent COVID-19 pandemic, the reliability of data to use for developing public health measures to control the diffusion of the virus was a major controversial issue, and there was no consensus about the true counts of cases and deaths of COVID-19 [34]. At the beginning, the only data were crude numbers shared on a daily basis by the National Health Authorities [35]; later on, more sources of data became available [36], making it more difficult for public health professionals to discern their level of reliability because of difficulties in estimating the actual rates, partially caused by the overload of the healthcare system, the avoidance of hospital care due to contagion fear and the absence of diagnoses due to dying at home or in nursing homes without a test and therefore lacking a microbiological diagnosis [37,38].

In this dishomogeneous setting, the methodology used in this study for data analysis based on a flow of data coming from Emergency Medical Service Activities proved to be an effective tool for monitoring infectious disease spread over time not only during the COVID-19 pandemic but also in cases of daily epidemiological oversight (e.g., for flu, heat waves or any other big event issue detection). Some real-world applications for this methodology may include the following:

- The early detection of any change in incidence of communicable diseases, possibly leading to overcoming the delay between the occurrence of cases and the publication of surveillance bulletins, especially at the beginning of an outbreak.
- The public health monitoring of any big event, such as Jubilee, when many people gather together and the onset of specific health issues may evolve in a very short period, making it difficult to detect them using classical surveillance methods based on swabs or blood testing.
- Governmental analysis and monitoring of both trends in incidence of the health event and pressure on the healthcare system during outbreaks to enhance optimal resource allocation and adaptation of the healthcare services in their response to the threats (e.g., pre-alerting hospitals to a possible increase in emergency case arrivals, adapting the number of hospital beds to respond to the possible increase in patients).

Furthermore, in a comparison of several such surveillance systems conducted in Britain [39], the authors concluded that attendances to emergency departments, whose precursors are emergency calls, were fairly accurate and timely epidemic trackers, confirming the usefulness of the approach presented in this paper.

In particular, as shown by Figure 1, high rates of potential patients of COVID-19 calling the EMS for respiratory and cardiological issues were already present at the beginning of 2020, long before the actual declaration of the pandemic by the WHO, thus showing that an infectious disease monitor system could be implemented through the EMS data. Moreover, in Figure 2, in addition to the already discussed COVID-19 potential patients corresponding to the peaks in pandemic waves in 2020–2022, there is also a clear representation of the peaks related to the seasonal flu. In agreement with different reports [40,41], the year with the strongest recent flu contribution was 2017, while the one with the weakest was 2016 when flu appears indeed almost absent from the time-series (Figure 2), confirming the usefulness of the AREU data collection system and of the analytical method used based on the EMS call excess estimation.

Figure 3 shows, on the other hand, the monitoring efficiency of AREU (measured in daily calls to the EMS) during the different pandemic waves. As shown, they mirrored the position of the official positive case peaks of the different COVID-19 waves, while the intensity of each peak depended on the efficiency of the official monitoring system in identifying the positive cases, which improved with time. Moreover, in comparing EMS calls to COVID-19 cases at the beginning of 2020, the graph shows a clear underestimation of COVID-19 cases, probably related to the lack of microbiological detection of COVID-19 at the start of the pandemic due to the poor availability of COVID-19 tests and the overwhelmed infectious disease monitoring system at that time. Later on, the graph shows a more pronounced, albeit still partial, overlap between the official monitoring system and the EMS data for the second and third waves of the COVID-19 pandemic, whilst, during the fourth wave, the intensity of the peaks reverses between EMS and COVID-19 cases, and the high peak of the latter might be explained by the implementation of the microbiological testing system, which highly increased the sensitivity of epidemiological monitoring.

In Figure 4, mortality data by ISTAT are compared to EMS calls. In the graph, it can be noted how the peaks relative to the different seasonal flu and COVID-19 waves are visible and match each other for the AREU and ISTAT data, showing a strong instantaneous correlation between calls to the EMS and the actual mortality rates, rising from high (r = 0.64) for seasonal flu to very high (r = 0.82) for COVID-19, with a discrepancy between the number of calls and ISTAT deaths being caused by two main reasons: (1) the fact that, fortunately, not all calls to the EMS result in patients’ deaths and (2) the lag between the start of the acute phase of illness and the actual death of the patient.

The top of Figure 5 indicates a stability over time in regard to patients’ ages for COVID-19 waves (top left) and flu (top right) in different years, while the histogram at the bottom shows that COVID-19 patients were, on average, younger than flu patients.

Figure 6 shows the comparison of the baseline-subtracted distributions for COVID-19, flu and baseline for sex, disease severity, hour of the call to the EMS, triage code, ambulance time to arrive at patient location and the return to hospital. Graphs again show good overlapping among the three different contributions for almost all variables, whereas the analysis for sex indicates that women were more affected by flu and less by COVID-19 than men.

Furthermore, the variables displayed in Figure 5 and Figure 6 show a potential, non-negligible discriminating power to distinguish baseline, flu and COVID-19 calls from each other. Each of the variables could be potentially combined in a neural network, AI-based approach. Preliminary tests have demonstrated that such a tool can be used to classify the type of call, i.e., predicting that a patient who called the 112-call center is affected by COVID-19, with high sensitivity and specificity.

Overall, our approach adds to previous work based on interrupted time-series regression conducted in another Italian region [42] which focused just on COVID-19, comparing the number of calls to emergency services in 2020–2021 with the number of calls made in 2019. Although their approach is not based on excess call estimation, the authors find a similar value for the correlation (r = 0.86) between daily deaths and symptomatic admissions. Another paper examined the number of calls in the Lombardy region in the first months of the pandemic in relation to the number of infected people [43] and found that the cross-correlation between them was maximized at a lag of 6 days with a lower but comparable value (about 0.8), which is consistent with the fact that these statistics were not based on the excess estimation and hence were likely to contain more noise.

Some limitations of this study include that it may be potentially affected by bias due to the choice of baseline subtraction, as this is inherent when applying excess estimation methods. However, as explained in previous work [26], the choice of a long baseline period (2015–2019) should provide a greater reliability to the excess estimate while simultaneously enabling us to separately analyze the signals of flu versus the signals of COVID-19. Another limitation, in regard to future epidemics, is the relatively short time-lag observed between the excess of emergency calls and deaths from COVID-19 which may, for some extreme health situations, be too short to enable changing their impact if appropriate resilience plans and resources are not already in place. Finally, the emergency call data only comprise few demographic variables and no biomarkers, which limits the power to discriminate between types of health threat.

## 5. Conclusions

We have analyzed the data of the Emergency Medical Service Activities of the Lombardy region, Italy, for the years 2015–2022, comparing them with the peaks of the COVID-19 pandemic and flu statistics. We showed that the excess of calls correlate strongly with the excess mortality derived from national statistics and that the different information contained in this emergency call dataset can be used to distinguish COVID-19 from flu patients. Future implementation of this monitoring system may be performed through machine learning (ML), i.e., selecting a set of variables which provide information on the emergency call and patient details and combining them in a dynamic predictive tool for early detection of new disease outbreaks and for monitoring the diffusion of infectious diseases, such as the presence of new pandemic waves.

In conclusion, the novel approach we introduced in this study, together with a possible future implementation using ML, could be an auxiliary tool for public health professionals to monitor the evolution of communicable diseases spreading among the general population as well as for detecting new disease outbreaks.

## Data Availability

Data are unavailable due to privacy restrictions related to Italian regulations regarding Emergency Medical Service Activities.

## References

[1] World Health Organization Timeline of WHO’s Response to COVID-19. https://www.who.int/emergencies/diseases/novel-coronavirus-2019/interactive-timeline.

[2] World Health Organization WHO Director-General’s Opening Remarks at the Media Briefing—5 May 2023. https://www.who.int/director-general/speeches/detail/who-director-general-s-opening-remarks-at-the-media-briefing---5-may-2023.

[3] Feehan J., Apostolopoulos V. (2021). Is COVID-19 the Worst Pandemic?. Maturitas.

[4] WHO Dashboard COVID-19. https://data.who.int/dashboards/covid19/cases.

[5] Presidenza del Consiglio dei Ministri (2020). Decreto Presidenza Consiglio Dei Ministri Del 9 Marzo 2020.

[6] Bezzini D., Schiavetti I., Manacorda T., Franzone G., Battaglia M.A. (2021). First Wave of COVID-19 Pandemic in Italy: Data and Evidence. Coronavirus Therapeutics—Volume II. Advances in Experimental Medicine and Biology.

[7] Porcu G., Chen Y.X., Bonaugurio A.S., Villa S., Riva L., Messina V., Bagarella G., Maistrello M., Leoni O., Cereda D. (2023). Web-Based Surveillance of Respiratory Infection Outbreaks: Retrospective Analysis of Italian COVID-19 Epidemic Waves Using Google Trends. Front. Public Health.

[8] García-Basteiro A.L., Chaccour C., Guinovart C., Llupià A., Brew J., Trilla A., Plasencia A. (2020). Monitoring the COVID-19 Epidemic in the Context of Widespread Local Transmission. Lancet Respir. Med..

[9] Mazick A. (2007). Workshop on mortality monitoring in Europe Monitoring Excess Mortality for Public Health Action: Potential for a Future European Network. Eurosurveillance.

[10] Nielsen J., Krause T.G., Mølbak K. (2018). Influenza-associated Mortality Determined from All-cause Mortality, Denmark 2010/11–2016/17: The FluMOMO Model. Influenza Other Respir. Viruses.

[11] Nielsen J., Vestergaard L.S., Richter L., Schmid D., Bustos N., Asikainen T., Trebbien R., Denissov G., Innos K., Virtanen M.J. (2019). European All-Cause Excess and Influenza-Attributable Mortality in the 2017/18 Season: Should the Burden of Influenza B Be Reconsidered?. Clin. Microbiol. Infect..

[12] Ceccarelli E., Minelli G., Egidi V., Jona Lasinio G. (2023). Assessment of Excess Mortality in Italy in 2020–2021 as a Function of Selected Macro-Factors. Int. J. Environ. Res. Public Health.

[13] Odone A., Delmonte D., Gaetti G., Signorelli C. (2021). Doubled Mortality Rate during the COVID-19 Pandemic in Italy: Quantifying What Is Not Captured by Surveillance. Public Health.

[14] Michelozzi P., de’Donato F., Scortichini M., Pezzotti P., Stafoggia M., De Sario M., Costa G., Noccioli F., Riccardo F., Bella A. (2020). Temporal Dynamics in Total Excess Mortality and COVID-19 Deaths in Italian Cities. BMC Public Health.

[15] Fior M., Mpampatsikos V. (2021). COVID-19 and Estimates of Actual Deaths in Italy. Scenarios for Urban Planning in Lombardy. J. Urban Manag..

[16] Scortichini M., Schneider dos Santos R., De’ Donato F., De Sario M., Michelozzi P., Davoli M., Masselot P., Sera F., Gasparrini A. (2021). Excess Mortality during the COVID-19 Outbreak in Italy: A Two-Stage Interrupted Time-Series Analysis. Int. J. Epidemiol..

[17] Kulu H., Dorey P. (2021). Infection Rates from COVID-19 in Great Britain by Geographical Units: A Model-Based Estimation from Mortality Data. Health Place.

[18] Kontis V., Bennett J.E., Rashid T., Parks R.M., Pearson-Stuttard J., Guillot M., Asaria P., Zhou B., Battaglini M., Corsetti G. (2020). Magnitude, Demographics and Dynamics of the Effect of the First Wave of the COVID-19 Pandemic on All-Cause Mortality in 21 Industrialized Countries. Nat. Med..

[19] Vanella P., Basellini U., Lange B. (2021). Assessing Excess Mortality in Times of Pandemics Based on Principal Component Analysis of Weekly Mortality Data—The Case of COVID-19. Genus.

[20] Modi C., Böhm V., Ferraro S., Stein G., Seljak U. (2021). Estimating COVID-19 Mortality in Italy Early in the COVID-19 Pandemic. Nat. Commun..

[21] Gil-Jardiné C., Chenais G., Pradeau C., Tentillier E., Revel P., Combes X., Galinski M., Tellier E., Lagarde E. (2021). Trends in Reasons for Emergency Calls during the COVID-19 Crisis in the Department of Gironde, France Using Artificial Neural Network for Natural Language Classification. Scand. J. Trauma. Resusc. Emerg. Med..

[22] Valent F., Licata S. (2020). Emergency Medical Services Calls During Italy’s COVID-19 Lockdown. Ann. Emerg. Med..

[23] Jaffe E., Sonkin R., Strugo R., Zerath E. (2021). Evolution of Emergency Medical Calls during a Pandemic—An Emergency Medical Service during the COVID-19 Outbreak. Am. J. Emerg. Med..

[24] Castro Delgado R., Delgado Sánchez R., Duque Del Río M.D.C., Arcos González P. (2021). Potential Capacity of an Emergency Dispatch Center to Predict COVID-19-Related Hospital and Intensive Care Unit Admissions. Emergencias.

[25] Scquizzato T., Landoni G., Ristagno G., Pruna A., Zangrillo A. (2023). Emergency Calls as an Early Indicator of Intensive Care Unit Demand for Coronavirus Disease 2019. Eur. J. Emerg. Med..

[26] del Re D., Palla L., Meridiani P., Soffi L., Loiudice M.T., Antinozzi M., Cattaruzza M.S. (2024). The Spread in Time and Space of COVID-19 Pandemic Waves: The Italian Experience from Mortality Data Analyses. Front. Public. Health.

[27] Areu Lombardia—Soreu. https://www.areu.lombardia.it/web/home/soreu.

[28] Fagoni N., Bellini L., Bonora R., Botteri M., Migliari M., Pagliosa A., Sechi G.M., Signorelli C., Zoli A., Stirparo G. (2024). Changing the Stroke Network during Pandemic Scenarios Does Not Affect the Management of Patients with a Positive Cincinnati Prehospital Stroke Scale. Neurol. Sci..

[29] Stirparo G., Bellini L., Ristagno G., Bonora R., Pagliosa A., Migliari M., Andreassi A., Signorelli C., Sechi G.M., Fagoni N. (2022). The Impact of COVID-19 on Lombardy Region ST-Elevation Myocardial Infarction Emergency Medical System Network—A Three-Year Study. J. Clin. Med..

[30] ISTAT—Mortalità. https://www.istat.it/notizia/dati-di-mortalita-cosa-produce-listat/.

[31] ROOT—Cern. https://root.cern/.

[32] Brun R., Rademakers F. (1997). ROOT—An Object Oriented Data Analysis Framework. Nucl. Instrum. Methods Phys. Res. A.

[33] Glasziou P.P., Sanders S., Hoffmann T. (2020). Waste in COVID-19 Research. BMJ.

[34] Lau H., Khosrawipour T., Kocbach P., Ichii H., Bania J., Khosrawipour V. (2021). Evaluating the Massive Underreporting and Undertesting of COVID-19 Cases in Multiple Global Epicenters. Pulmonology.

[35] Istituto Superiore Di Sanità Dati Della Sorveglianza Integrata COVID-19 in Italia. https://www.epicentro.iss.it/coronavirus/sars-cov-2-dashboard.

[36] Alamo T., Reina D., Mammarella M., Abella A. (2020). COVID-19: Open-Data Resources for Monitoring, Modeling, and Forecasting the Epidemic. Electronics.

[37] Rizzo M., Foresti L., Montano N. (2020). Comparison of Reported Deaths From COVID-19 and Increase in Total Mortality in Italy. JAMA Intern. Med..

[38] Mannucci E., Nreu B., Monami M. (2020). Factors Associated with Increased All-Cause Mortality during the COVID-19 Pandemic in Italy. Int. J. Infect. Dis..

[39] Brainard J., Lake I.R., Morbey R.A., Jones N.R., Elliot A.J., Hunter P.R. (2023). Comparison of Surveillance Systems for Monitoring COVID-19 in England: A Retrospective Observational Study. Lancet Public Health.

[40] Fattore G., Pongiglione B., Vezzosi L. (2024). Excess Hospitalizations and In-Hospital Mortality Associated with Seasonal Influenza in Italy: A 11-Year Retrospective Study. BMC Infect. Dis..

[41] Rosano A., Bella A., Gesualdo F., Acampora A., Pezzotti P., Marchetti S., Ricciardi W., Rizzo C. (2019). Investigating the Impact of Influenza on Excess Mortality in All Ages in Italy during Recent Seasons (2013/14–2016/17 Seasons). Int. J. Infect. Dis..

[42] Vinci A., Pasquarella A., Corradi M.P., Chatzichristou P., D’Agostino G., Iannazzo S., Trani N., Parafati M.A., Palombi L., Ientile D.A. (2022). Emergency Medical Services Calls Analysis for Trend Prediction during Epidemic Outbreaks: Interrupted Time Series Analysis on 2020–2021 COVID-19 Epidemic in Lazio, Italy. Int. J. Environ. Res. Public Health.

[43] Rivieccio B.A., Micheletti A., Maffeo M., Zignani M., Comunian A., Nicolussi F., Salini S., Manzi G., Auxilia F., Giudici M. (2021). COVID-19, Learning from the Past: A Wavelet and Cross-Correlation Analysis of the Epidemic Dynamics Looking to Emergency Calls and Twitter Trends in Italian Lombardy Region. PLoS ONE.

