# Correction: del Re et al. Data from Emergency Medical Service Activities: A Novel Approach to Monitoring COVID-19 and Other Infectious Diseases. *Diagnostics* 2025, *15*, 181

**DOI:** 10.3390/diagnostics15060745

**Published:** 2025-03-17

**Authors:** Daniele del Re, Luigi Palla, Paolo Meridiani, Livia Soffi, Michele Tancredi Loiudice, Martina Antinozzi, Maria Sofia Cattaruzza

**Affiliations:** 1 Department of Physics, Sapienza University of Rome, 00185 Rome, Italy; daniele.delre@uniroma1.it; 2 Department of Public Health and Infectious Diseases, Sapienza University of Rome, 00185 Rome, Italy; mariasofia.cattaruzza@uniroma1.it; 3 INFN Istituto Nazionale Fisica Nucleare, Sezione di Roma, 00146 Rome, Italy; paolo.meridiani@roma1.infn.it (P.M.); livia.soffi@roma1.infn.it (L.S.); 4 Department of Developmental and Social Psychology, Faculty of Medicine and Psychology, Sapienza University of Rome, 00185 Rome, Italy; michele.loiudice@uniroma1.it

## Error in Figure

In the original publication [1], there was a mistake in Figure 5 as published. The top right panel representing the pattern of patients’ age for flu during three pre-COVID-19 years was displayed twice, so the left panel has been amended to represent the age patterns of three COVID-19 waves. The corrected Figure 5 appears below. The authors state that the scientific conclusions are unaffected. This correction was approved by the Academic Editor. The original publication has also been updated.

## Figures and Tables

**Figure 5:**
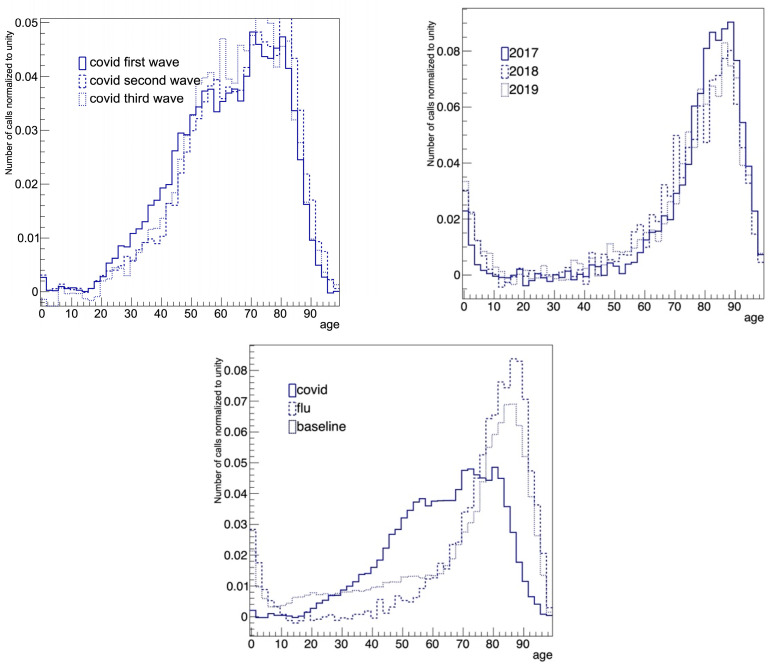
**Top**: Comparison of the baseline-subtracted distributions of the age of the patients for (**left**) the three COVID-19 waves and (**right**) flu for different years. **Bottom**: Comparison of the baseline-subtracted distributions for COVID-19 (period 3), flu (period 2) and baseline (period 1).
